# Quantifying cause-related mortality by weighting multiple causes of death

**DOI:** 10.2471/BLT.16.172189

**Published:** 2016-10-11

**Authors:** Clara Piffaretti, Margarita Moreno-Betancur, Agathe Lamarche-Vadel, Grégoire Rey

**Affiliations:** a CépiDc–Inserm, Epidemiology Centre on Medical Causes of Death, 80, rue du Général Leclerc, 94276 le Kremlin-Bicêtre, Cedex, France.; b Clinical Epidemiology and Biostatistics Unit, Murdoch Childrens Research Institute, Melbourne, Australia.

## Abstract

**Objective:**

To investigate a new approach to calculating cause-related standardized mortality rates that involves assigning weights to each cause of death reported on death certificates.

**Methods:**

We derived cause-related standardized mortality rates from death certificate data for France in 2010 using: (i) the classic method, which considered only the underlying cause of death; and (ii) three novel multiple-cause-of-death weighting methods, which assigned weights to multiple causes of death mentioned on death certificates: the first two multiple-cause-of-death methods assigned non-zero weights to all causes mentioned and the third assigned non-zero weights to only the underlying cause and other contributing causes that were not part of the main morbid process. As the sum of the weights for each death certificate was 1, each death had an equal influence on mortality estimates and the total number of deaths was unchanged. Mortality rates derived using the different methods were compared.

**Findings:**

On average, 3.4 causes per death were listed on each certificate. The standardized mortality rate calculated using the third multiple-cause-of-death weighting method was more than 20% higher than that calculated using the classic method for five disease categories: skin diseases, mental disorders, endocrine and nutritional diseases, blood diseases and genitourinary diseases. Moreover, this method highlighted the mortality burden associated with certain diseases in specific age groups.

**Conclusion:**

A multiple-cause-of-death weighting approach to calculating cause-related standardized mortality rates from death certificate data identified conditions that contributed more to mortality than indicated by the classic method. This new approach holds promise for identifying underrecognized contributors to mortality.

## Résumé

**Objectif:**

Étudier une nouvelle approche permettant de calculer des taux comparatifs de mortalité due à différentes causes en pondérant chaque cause de décès déclarée sur les certificats de décès.

**Méthodes:**

Nous avons calculé des taux comparatifs de mortalité due à différentes causes à partir des données de certificats de décès émis en France en 2010 suivant: (i) la méthode classique, où nous avons uniquement tenu compte de la cause sous-jacente de décès; et (ii) trois nouvelles méthodes de pondération de causes multiples de décès, qui consistaient à appliquer une pondération aux différentes causes de décès mentionnées sur les certificats de décès: les deux premières méthodes tenant compte de plusieurs causes de décès consistaient à appliquer une pondération autre que zéro à toutes les causes mentionnées et la troisième consistait à appliquer une pondération autre que zéro uniquement à la cause sous-jacente et à d'autres causes aggravantes, extérieures au processus pathologique principal. La somme des pondérations pour chaque certificat de décès était de 1. Ainsi, chaque décès avait la même influence sur les estimations de la mortalité, sans changer le nombre total de décès. Les taux de mortalité obtenus suivant ces différentes méthodes ont ensuite été comparés.

**Résultats:**

En moyenne, 3,4 causes étaient mentionnées sur chaque certificat de décès. Le taux comparatif de mortalité calculé selon la troisième méthode de pondération de causes multiples de décès était plus de 20% supérieur à celui calculé selon la méthode classique pour cinq catégories de maladies: maladies de la peau, troubles mentaux, maladies endocriniennes et nutritionnelles, maladies du sang et maladies uro-génitales. En outre, cette méthode a mis en relief la charge de mortalité associée à certaines maladies dans des groupes d'âge spécifiques.

**Conclusion:**

L'approche consistant à pondérer des causes multiples de décès afin de calculer des taux comparatifs de mortalité due à différentes causes à partir des données figurant sur des certificats de décès a permis de repérer les pathologies qui contribuaient plus à la mortalité que ce qu'indiquait la méthode classique. Cette nouvelle approche devrait permettre d'identifier les facteurs peu reconnus qui contribuent pourtant à la mortalité.

## Resumen

**Objetivo:**

Investigar un nuevo enfoque para calcular las tasas estandarizadas de mortalidad relacionada con las causas que implique la asignación de variables de cada causa de muerte indicada en los certificados de defunción.

**Métodos:**

Se derivaron las tasas estandarizadas de mortalidad relacionada con las causas de certificados de defunción en Francia en 2010 utilizando: (i) el método clásico, que consideraba únicamente la causa subyacente de la muerte; y (ii) tres nuevos métodos de evaluación de múltiples causas de muerte, que asignaban variables a varias causas de muerte mencionadas en los certificados de defunción: los primeros dos métodos de múltiples causas de muerte asignaron variables no nulas en todas las causas mencionadas y el tercero asignó las mismas variables sólo a la causa subyacente y otras causas contribuyentes que no formaban parte del proceso mórbido principal. Dado que la suma de las variables de cada certificado era 1, cada defunción tenía la misma influencia en las estimaciones de mortalidad y el número total de muertes permaneció intacto. Se compararon las tasas de mortalidad derivadas utilizando los distintos métodos.

**Resultados:**

De media, cada certificado enumeraba 3,4 causas por muerte. La tasa de mortalidad estandarizada calculada utilizando el tercer método de evaluación de múltiples causas de muerte fue más de un 20% superior a la calculada utilizando el método clásico para cinco categorías de enfermedades: enfermedades cutáneas, trastornos mentales, enfermedades endocrinas y nutricionales, enfermedades sanguíneas y enfermedades genitourinarias. Asimismo, este método destacó el umbral de mortalidad relacionado con determinadas enfermedades de grupos de edades en particular.

**Conclusión:**

Un enfoque de evaluación de múltiples causas de muerte para calcular las tasas estandarizadas de mortalidad relacionada con las causas de datos recopilados de certificados de defunción identificó las condiciones que contribuyeron más a la mortalidad que las indicadas en el método clásico. Este nuevo enfoque promete identificar contribuyentes no reconocidos a la mortalidad.

## ملخص

**الغرض:**

الاستقصاء عن نهج جديد يختص بحساب معدلات الوفيات القياسية المرتبطة بسبب الوفاة والذي يتضمن تخصيص الترجيحات لكل سبب من أسباب الوفاة المدونة في شهادات الوفاة.

**الطريقة:**

قمنا باشتقاق معدلات الوفيات القياسية المرتبطة بسبب الوفاة من بيانات شهادة الوفاة في فرنسا في عام 2010 باستخدام التالي: (أ) الطريقة القديمة، والتي أخذت في الاعتبار فقط السبب الدفين لحدوث الوفاة؛ و(ب) ثلاث وسائل جديدة لترجيح أسباب الوفاة المتعددة، والتي عملت على تخصيص الترجيحات للعديد من أسباب الوفاة المذكورة في شهادات الوفاة: قامت أول وسيلتين لترجيح أسباب الوفاة المتعددة بتخصيص ترجيحات غير صفرية لجميع الأسباب المذكورة، فيما قامت الوسيلة الثالثة بتخصيص ترجيحات غير صفرية لسبب الوفاة الدفين فقط والأسباب الأخرى المساهمة في حدوث الوفاة والتي لم تكن جزءًا من الحالة المرضية الرئيسية. وبينما كان مجموع الترجيحات لكل شهادة وفاة يساوي 1، فإن كل حالة وفاة كان لديها تأثير متساوٍ على تقديرات الوفيات ولم يتغير العدد الإجمالي لحالات الوفاة. وتمت مقارنة معدلات الوفيات التي تم اشتقاقها باستخدام مختلف الوسائل.

**النتائج:**

تم في المتوسط إدراج 3.4 أسباب لكل وفاة في كل شهادة وفاة صادرة. كان معدل الوفيات القياسي الذي تم حسابه باستخدام الوسيلة الثالثة لترجيح أسباب الوفاة المتعددة أعلى بنسبة تزيد عن 20‏% عن المعدل الذي تم حسابه باستخدام الوسيلة القديمة بالنسبة للخمس فئات التالية من الأمراض: الأمراض الجلدية، والاضطرابات الذهنية، وأمراض الغدد الصماء والأمراض المرتبطة بالتغذية، وأمراض الدم، وأمراض الجهاز البولي التناسلي. وعلاوة على ذلك، أبرزت هذه الوسيلة عبء معدلات الوفيات المرتبط بأمراض معينة في فئات عمرية محددة.

**الاستنتاج:**

كان نهج ترجيح أسباب الوفاة المتعددة بغرض حساب معدلات الوفيات القياسية المرتبطة بأسباب الوفاة الصادرة عن بيانات شهادات الوفاة قد ساهم في تحديد الظروف التي أدت بشكلٍ أكبر في حدوث الوفيات عن الظروف التي تشير إليها الطريقة القديمة. ويبشر هذا النهج الجديد بالخير في تحديد العوامل المساهمة غير المعروفة في حدوث الوفيات.

## 摘要

**目的:**

旨在调查新的计算因果关联标准化死亡率的方法，该方法涉及到为死亡证明上报告的每种死因分配权重。

**方法:**

我们使用以下方法从 2010 年法国死亡证明数据中得出因果关联的标准化死亡率： (i) 传统方法，仅考虑根本死因；和 (ii) 三种新颖的多重死因加权平均法，该方法为死亡证明上提到的多重死因分配权重： 前两种多重死因方法将非零权重分配至所有涉及原因，第三种方法仅将非零权重分配给根本原因以及其他不构成主要病变过程的间接原因。 由于每个死亡证明的权重总和为 1，所以每例死亡对死亡率估值具有同等影响，而且死亡总数未变。 我们比较了使用不同方法得出的死亡率。

**结果:**

平均每张证明上每例死亡列出 3.4 种原因。 在下列五种疾病类别中，使用第三种多重死因加权平均方法计算出的标准化死亡率比使用传统方法计算出的死亡率高出 20% 以上：皮肤疾病、精神障碍、内分泌和营养性疾病、血液疾病和泌尿生殖系统疾病。 此外，此方法着重强调了与特定年龄段某些疾病相关的死亡率负担。

**结论:**

与传统方法相比，通过多重死因加权平均法从死亡证明数据中计算出的因果关联死亡率，能够确定出对死亡率影响更大的因素。 该新方法有望实现对未发现死亡原因的确定。

## Резюме

**Цель:**

Изучить новый подход к расчету стандартизированных показателей смертности по определенным причинам, включающий присваивание весового коэффициента каждой причине смерти, указанной в свидетельстве о смерти.

**Методы:**

На основании данных, внесенных в свидетельства о смерти во Франции в 2010 году, авторы вычислили показатели смертности по определенным причинам (I) классическим методом, который учитывал только причину, непосредственно приведшую к смерти, и (ii) тремя новыми методами с учетом весовых коэффициентов для нескольких причин смерти, в которых нескольким причинам смерти, указанным в свидетельстве о смерти, присваивались весовые коэффициенты: первые два таких метода предусматривали присвоение отличных от нуля весовых коэффициентов всем названным в свидетельстве причинам смерти, а третий предусматривал отличные от нуля весовые коэффициенты только для непосредственной причины смерти и для других причин, которые не были частью основного процесса, приведшего к смерти пациента. Так как сумма весовых коэффициентов для каждого свидетельства о смерти была равна единице, каждый случай смерти одинаковым образом влиял на оценку смертности, а общее количество смертей осталось неизменным. Для полученных таким образом показателей смертности затем было проведено сравнение.

**Результаты:**

В среднем в каждом свидетельстве о смерти были указаны 3,4 причины смерти. Стандартизированные показатели смертности, вычисленные третьим методом с учетом весовых коэффициентов, более чем на 20% превышали показатели, рассчитанные классическим методом, для пяти категорий заболеваний: болезней кожи, нарушений психического здоровья, болезней эндокринной системы и расстройств питания, заболеваний крови и болезней мочеполовой системы. Более того, этот метод позволил выявить долю смертности, ассоциируемую с определенными возрастными группами.

**Вывод:**

Подход, согласно которому при расчете стандартизированных показателей смертности по определенной причине на основании данных, указанных в свидетельствах о смерти, предлагается учитывать весовые коэффициенты различных причин смерти, позволил выявить заболевания и состояния, которые в большей мере вносят вклад в смертность, нежели это демонстрировал стандартный метод определения таких показателей. Этот новый подход обещает выявить дополнительные неизвестные причины смертности.

## Introduction

Good understanding of mortality data is essential for developing and evaluating health policies. The causes of any death are usually reported on parts I and II of a death certificate, in accordance with the international form presented in the *International classification of diseases and related health problems, tenth revision* (ICD-10),^1^ and data are usually collected in a standardized and consistent way.^2^ In part I, the physician describes the causal sequence of events that led directly to the death. In part II, the physician can report any other significant morbid condition but only if that condition may have contributed to the death.

Generally, cause-of-death statistics are derived from the so-called underlying cause of death in a process hereafter referred to as the classic method.^3^ The World Health Organization (WHO) defines the underlying cause of death as “the disease or injury which initiated the train of morbid events leading directly to death or the circumstances of the accident or violence which produced the fatal injury”.^1^ However, deaths are often caused by more than one disease. Moreover, in a world characterized by an ageing population and decreasing mortality and fertility, death due to infectious disease is progressively being replaced by death due to chronic and degenerative diseases.^4^^–^^6^ As a result, the classic method discards potentially useful information about the contribution of other conditions to a death.

Today, analysis of mortality data increasingly uses a multiple-cause-of-death approach,^3^^,^^4^^,^^7^^–^^12^ which is defined as any statistical treatment that simultaneously considers more than one of the causes of death reported on a death certificate. In particular, such approaches have been used to recalculate mortality attributable to specific conditions. In practice, when cause-specific mortality is re-evaluated to take into account multiple causes of death, the number of mentions of a specific cause is usually considered – here the statistical unit is the cause of death rather than the death itself, which raises serious questions about interpretation. For example, studies examining the influence of several diseases on mortality may count a single death two or more times if two or more causes of death are mentioned on the certificate. The resulting apparent increase in mortality could yield an artificial increase in statistical power and possibly result in misleading inferences. An additional problem is that each cause of death mentioned on a certificate is given an equal weight, even though its individual contribution may not have been equally important – the relative importance of each cause of death is not considered.

In this study, we investigated an experimental approach that assigns a weight to each cause of death listed on a death certificate by analysing French death certificate data using three multiple-cause-of-death weighting methods. This approach conceptualizes death as the outcome of a mixture of conditions, as we described elsewhere.^13^ Consequently, each death contributes only a fraction, rather than a unit, when calculating standardized mortality rates for each cause of death – the fraction depends on the weight assigned. The approach accepts that multiple factors may contribute to a death but also reflects the relative contribution of each cause of death.^13^ Use of a multiple-cause-of-death weighting approach could help us identify conditions whose contribution to mortality is underestimated by the classic method.

## Methods

We examined data on all deaths reported in France during 2010. We had access to information on all the causes of death declared on death certificates, including the underlying cause of death, as coded using the ICD-10 by CépiDc–Inserm– the epidemiology centre on medical causes of death of the French National Institute for Health and Medical Research. We used the 2012 version of the European shortlist for causes of death to analyse mortality by cause-of-death category,^14^ though the list was modified slightly for the analysis. In addition, we removed codes for causes of death that were not relevant to our study, such as those that did not refer to diseases but rather to: (i) risk factors; (ii) family history; (iii) socioeconomic and psychosocial circumstances; and (iv) injury or poisoning or other external causes of death (i.e. ICD-10 cause-of-death codes beginning with S, T, U or Z, which relate to chapters XIX, XXI and XXII). Of note, none of these causes was designated an underlying cause of death.

First, we classified the data using cause-of-death categories and determined whether each cause was reported as an underlying or a contributory cause. We also examined the number of causes reported on each death certificate, whether in both parts of the certificate or only in part II. Then we calculated age- and sex-standardized mortality rates for each cause-of-death category using: (i) the classic method, which considered only the underlying cause of death; and (ii) three multiple-cause-of-death weighting methods that assigned a weight to each cause of death, as described below. For the analysis, we used the Eurostat Europe and European Free Trade Association standard population for 2013.^15^ All analyses were performed using SAS v. 9.3 (SAS Institute Inc., Cary, United States of America).

### Multiple-cause weighting

The first multiple-cause-of-death weighting method, MCW_1_, attributes an equal weight to each cause of death reported on a death certificate. Thus, if cause *i* is mentioned on certificate *i,*on which a total of *n_i_* causes are reported, the weight attributed to cause *c, w_c,i _* is given by: 


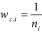
(1)

Here, the underlying cause is not given a greater weight than other causes.

The second weighting method, MCW_2_, attributes a weight *w^UC^* to the disease selected as the underlying cause of death, with *w^UC^* having a fixed value between 0 and 1. The total remaining weight (i.e. 1 – *w^UC^*) is distributed among all other causes of death mentioned on the certificate (i.e. *n_i_* – 1). Hence, the weight attributed to cause *c* on certificate *i*, 

*w_c,i _* , is given by:
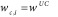
(2)if *c* is the underlying cause, and by:
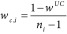
(3)if *c* is the not underlying cause.

With the classic method, *w^UC^*=1, the death is wholly attributed to the underlying cause regardless of other causes mentioned on the certificate. In contrast, the first two weighting methods enable all diseases mentioned on the death certificate to be included in the analysis. Although the attributed value of *w^UC ^*is subjective, so is choosing *w^UC^* to be 1. Therefore, the effect of different choices of *w^UC^* should be examined in a sensitivity analysis. In our analysis, we set *w^UC ^*equal to 0.5 to give a good illustration of the impact of the weighting method on standardized mortality rates. Choosing an intermediate weight between 0.5 and 1 would lead to mortality rates between those based on the classic method and those based on a weighting method with *w^UC^* set to 0.5.

The third weighting method, MCW_3_, is similar to the second except that all causes of death mentioned in part I of the death certificate other than the underlying cause are given a weight of zero. Hence, the weight attributed to cause *c* on certificate *i*, *w_c,i _*is given by:
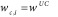
(4)if *c* is the underlying cause, by:
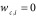
(5)if *c* is mentioned in part I and is not the underlying cause, and by: 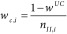
(6)if *c* is mentioned in part II and is not the underlying cause, where *w^UC^* is the weight attributed to the underlying cause of death and *n_II,i_* is the number of causes reported on part II of the death certificate (apart from the underlying cause if it is reported on part II, as could occur with some ICD-10 coding rules). The aim of this approach was to take into account the underlying cause of death and only other causes of death that were regarded as being on a different causal pathway from the main morbid process initiated by the underlying cause. Studying separate disease processes in this way is more meaningful from a causal perspective.

For both MCW_2_ and MCW_3 _methods, when only one cause is reported, that cause is necessarily the underlying cause and its weight *w_c,i_* is 1. In addition, with all three weighting methods, the sum of the weights for all the different causes of death on each death certificate is 1. Moreover, the sum of the weights across individuals equals the total number of deaths. Consequently, each death has an equal influence on mortality estimates. Table 1 illustrates how the classic method and the three weighting methods are applied (additional examples are available from the corresponding author on request).

**Table 1:** Weights applied to causes of death on a death certificate calculation method

Cause of death on death certificate	Weights applied to causes of death			
	Classic method^a^	Multiple-cause-of-death weighting method^b^		
		MCW_1_	MCW_2_	MCW_3_
**Part I**				
a. Pneumonia	0	1/5 = 0.2	0.5/4 = 0.125	0
b. Chronic respiratory failure	0	1/5 = 0.2	0.5/4 = 0.125	0
c. Chronic obstructive pulmonary disease^c^	1	1/5 = 0.2	*w^UC^* = 0.5	*w^UC^* = 0.5
d. No cause listed	NA	NA	NA	NA
**Part II**				
Diabetes	0	1/5 = 0.2	0.5/4 = 0.125	0.5/2 = 0.25
Dementia	0	1/5 = 0.2	0.5/4 = 0.125	0.5/2 = 0.25

MCW: Multiple-cause-of-death weighting method; NA: not applicable; *w^UC^*: weight attributed to the underlying cause of death.

^a^ With the classic method, only the underlying cause of death specified on the death certificate is considered when calculating mortality rates.

^b^ Details of the three multiple-cause-of-death weighting methods, MCW_1_, MCW_2_ and MCW_3_ are given in the main text.

^c^ Underlying cause of death mentioned on the death certificate.

After we assigned weights to each cause of death on each death certificate using a weighting method, we calculated age- and sex-standardized mortality rates for each cause. First, the sum of the weights attributed to cause *c* mentioned on death certificates across all individuals *i* was computed for specific age (*a*) and sex (*s*) groups:
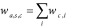
(7)where *w_c,i_* is the weight attributed to cause *c* on the certificate of individual i. Then, the standardized mortality rate for cause *c* was obtained as:
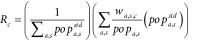
(8)where *R_c_* is the standardized mortality rate, 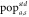
(9)and *pop_a,s_* are the number of individuals of age *a* and sex *s* (by 5-year age group and sex) in the standard population and in the French population,^16^ respectively. Finally, for each cause of death, we calculated the change in the standardized mortality rate derived using each weighting method relative to the corresponding rate obtained using the classic method, both overall and by age group and sex.

## Results

**Fig. 1:**
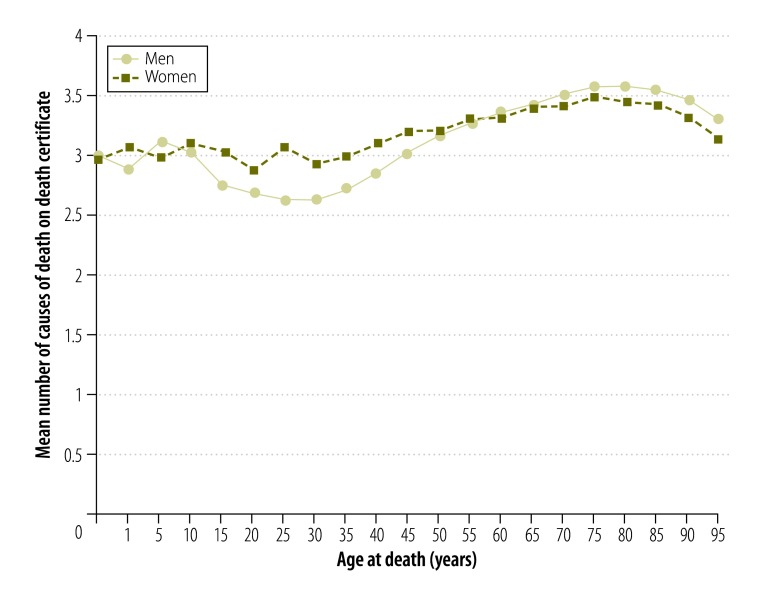
Number of causes of death on each death certificate, by age and sex, 2010, France

Note: The mean number of causes of death was computed by the following age-groups: 0–11 months, 12–23 months, 2–5 years, 5–9 years, 10–14 years, 15–19 years, etc.

In total, 552 571 deaths were reported in France in 2010. On average, 3.4 causes of death were mentioned on each death certificate (standard deviation: 1.92; median: 3; interquartile range: 2 to 4). The variation in the mean number of causes of death by age was low: it varied between 3.2 and 3.6 per individual over the age range 55 to 93 years, within which 80% of deaths occurred (Fig. 1). However, the mean was lower in individuals aged 15 to 35 years, varying between 2.6 and 3.1 causes in each certificate. Some categories of the underlying cause of death appeared more frequently than others on certificates that mentioned a high number of causes: a high mean number of causes was associated with conditions in the categories of *musculoskeletal diseases*, *skin diseases*, *endocrine and nutritional diseases* and *blood diseases* (Table 2). Moreover, when one of these conditions was mentioned as the underlying cause of death, the ratio of the number of mentions of the condition to the number of mentions as the underlying cause was also high. However, the category *symptoms, signs, ill-defined causes* was associated with the highest ratio and with the lowest mean number of causes reported.

**Table 2:** Causes of death mentioned on death certificates, 2010, France

Cause-of-death category^a^	Total no. of mentions of cause on death certificates^b^	No. of mentions of cause as underlying cause of death on certificates^b^	Ratio of no. of mentions of cause to no. of mentions as underlying cause	Mean no. of all causes of death per certificate
Musculoskeletal diseases	11 692	3 744	3.12	4.58
Skin diseases	10 506	1 459	7.20	4.37
Endocrine and nutritional diseases	87 782	20 069	4.37	4.27
Blood diseases	14 957	2 313	6.47	4.17
Digestive system diseases	71 738	23 954	2.99	3.97
Genitourinary diseases	49 293	9 979	4.94	3.90
Infectious diseases	48 977	11 129	4.40	3.88
Congenital malformations	3 072	1 548	1.98	3.78
Mental disorders	65 044	18 265	3.56	3.70
External causes of morbidity and mortality	50 000	38 671	1.29	3.65
Respiratory system diseases	140 936	32 640	4.32	3.55
Circulatory system diseases	442 166	146 057	3.03	3.49
Nervous system diseases	73 247	32 850	2.23	3.48
Pregnancy or childbirth complications	149	74	2.01	3.47
Neoplasms	308 445	162 113	1.90	3.43
Perinatal conditions	5 137	1 457	3.53	3.33
Symptoms, signs, ill-defined causes	353 068	35 356	9.99	1.40
Other	124 889	NA	NA	NA

NA: not applicable.

^a^ The cause-of-death categories are those listed in the *European shortlist for causes of death*, *2012*.^14^

^b^ In total, 552 571 deaths were reported in France in 2010.

Here, we report mainly our findings with the MCW_3_ method, which are the easiest to interpret and the most interesting. We found that the increase in the standardized mortality rate derived using this method relative to the classic method exceeded 20% in five cause-of-death categories: *skin diseases*, *mental disorders*, *endocrine and nutritional diseases*, *blood diseases* and *genitourinary diseases*(Table 3). The overall increase in the standardized mortality rate we observed for *mental disorders* was due in a large part to increases in specific subcategories: for *other mental and behavioural disorders* the increase was 112% and for *alcohol abuse* (including alcoholic psychosis), it was 43% (Table 4; available at: http://www.who.int/bulletin/volumes/94/121/16-172189). In contrast, the increase for *drug dependence and toxicomania* was 28% and for *dementia*, 12%. Notable increases were also observed in other disease subcategories: *rheumatoid arthritis and osteoarthrosis* increased by 44%, *other diseases of the circulatory system* by 19% and *viral hepatitis* by 19%. There was either no change or a small decrease in the standardized mortality rate in categories such as *diseases of the circulatory system*, *diseases* *of the* *respiratory system *and *perinatal diseases*. However, as expected, our analysis found a decrease in the contribution of conditions that are almost systematically specified as the underlying cause of death: for example, *external causes of morbidity and mortality*, *neoplasms*, *congenital malformations* and *digestive system diseases*. These decreases were most marked with the MCW_1_ method (Table 3), particularly when the number of other causes of death mentioned was high, because this method does not attribute a greater weight to the underlying cause relative to other causes.

**Table 3:** Standardized cause-related mortality rates, by calculation method and cause-of-death category, 2010, France

Cause-of-death category^a^	Standardized mortality derived using the classic method,^b^ per 100 000 population	Change in standardized mortality with the multiple-cause-of-death weighting method^c^ relative to the classic method, %		
		MCW_1_	MCW_2_	MCW_3_
Infectious diseases	19.2	22	16	−14
External causes of morbidity and mortality	65.2	−40	−31	−13
Digestive system diseases	40.8	−15	−10	−11
Neoplasms	287.0	−36	−26	−7
Respiratory system diseases	59.3	22	15	−5
Circulatory system diseases	250.1	−9	−8	−1
Perinatal conditions	1.8	7	6	2
Pregnancy or childbirth complications	0.1	−31	−22	3
Congenital malformations	2.2	−41	−31	4
Nervous system diseases	53.1	−29	−23	5
Musculoskeletal diseases	6.2	−28	−20	11
Symptoms, signs, ill-defined causes	58.4	253	194	15
Genitourinary diseases	18.0	26	15	24
Blood diseases	3.9	60	40	26
Endocrine and nutritional diseases	33.7	3	6	33
Mental disorders	30.1	2	0	34
Skin diseases	2.4	69	44	42

MCW: Multiple-cause-of-death weighting method.

^a^ The cause-of-death categories are those listed in the *European shortlist for causes of death, 2012*.^14^

^b^ With the classic method, standardized mortality was calculated using only the underlying cause of death specified on the death certificate.

^c^ Details of the three multiple-cause-of-death weighting methods, MCW_1_, MCW_2_ and MCW_3_, are given in the main text.

**Table 4:** Standardized cause-related mortality rates, by calculation method and cause-of-death subcategory, 2010, France

Cause-of-death category and subcategory^a^	Standardized mortality derived using the classic method,^b^ per 100 000 population	Change in standardized mortality with the multiple-cause-of-death weighting method^c^ relative to the classic method, %		
		MCW_1_	MCW_2_	MCW_3_
**Infectious and parasitic diseases**				
Tuberculosis	1.1	−45	−32	1
AIDS (HIV disease)	0.8	−49	−25	−4
Viral hepatitis	1.1	0	2	19
Other infectious and parasitic diseases	16.2	31	23	−17
**Neoplasms**				
Malignant neoplasm of lip, oral cavity, pharynx	7.4	−53	−40	−9
Malignant neoplasm of oesophagus	7.3	−55	−41	−10
Malignant neoplasm of stomach	8.6	−57	−43	−9
Malignant neoplasm of colon, rectum and anus	30.1	−60	−44	−9
Malignant neoplasm of liver and intrahepatic bile ducts	14.9	−55	−41	−10
Malignant neoplasm of pancreas	16.1	−54	−41	−9
Malignant neoplasm of larynx	2.3	−51	−39	−6
Malignant neoplasm of trachea, bronchus, lung	54.7	−56	−42	−10
Malignant melanoma of skin	3.1	−63	−46	−7
Malignant neoplasm of breast	17.7	−61	−44	−5
Malignant neoplasm of cervix uteri	1.2	−60	−44	−7
Malignant neoplasm of other and unspecified parts of uterus	3.5	−58	−43	−7
Malignant neoplasm of ovary	5.2	−61	−45	−7
Malignant neoplasm of prostate	21.4	−54	−40	−4
Malignant neoplasm of kidney	6.3	−61	−45	−9
Malignant neoplasm of bladder	9.9	−57	−42	−9
Malignant neoplasm of brain and central nervous system	5.9	−44	−35	−6
Malignant neoplasm of thyroid	0.6	−55	−41	−4
Hodgkin’s disease and lymphomas	8.3	−49	−37	−8
Leukaemia	9.9	−50	−38	−7
Other malignant neoplasm of lymphoid and haematopoietic tissue	5.4	−52	−39	−8
Other malignant neoplasms	35.4	103	81	4
Nonmalignant neoplasms (benign and uncertain)	11.7	−40	−31	−4
**Diseases of the blood**	3.9	60	40	26
**Endocrine, nutritional and metabolic diseases**				
Diabetes mellitus	19.2	−25	−9	30
Other endocrine, nutritional and metabolic diseases	14.5	39	25	38
**Mental and behavioural disorders**				
Dementia	19.4	−33	−25	12
Alcohol abuse (including alcoholic psychosis)	5.2	10	2	43
Drug dependence, toxicomania	0.3	−9	−8	28
Other mental and behavioural disorders	5.1	124	92	112
**Diseases of the nervous system and sense organs**				
Parkinson disease	8.9	−42	−32	6
Alzheimer disease	28.0	−42	−33	5
Other diseases of the nervous system and sense organs	16.3	1	−1	6
**Diseases of the circulatory system**				
Ischaemic heart diseases: acute myocardial infarction	31.1	−50	−38	−17
Other ischaemic heart diseases	33.1	−41	−30	−4
Other heart diseases	87.2	24	16	1
Cerebrovascular diseases	54.5	−32	−25	−11
Other diseases of the circulatory system	44.3	7	4	19
**Diseases of the respiratory system**				
Influenza	0.2	−64	−43	−20
Pneumonia	19.2	10	5	−13
Asthma	1.6	−32	−25	8
Other chronic lower respiratory diseases	15.6	−41	−29	−2
Other diseases of the respiratory system	22.7	80	58	−1
**Diseases of the digestive system**				
Ulcer of stomach, duodenum and jejunum	1.6	−47	−27	−15
Cirrhosis, fibrosis and chronic hepatitis	12.8	−44	−29	−6
Other diseases of the digestive system	26.4	1	1	−13
**Diseases of the skin and subcutaneous tissue**	2.4	69	44	42
**Diseases of the musculoskeletal system or connective tissue**				
Rheumatoid arthritis and osteoarthrosis	0.8	−11	−8	44
Other diseases of the musculoskeletal system or connective tissues	5.3	−30	−22	6
**Diseases of the genitourinary system**				
Diseases of kidney and ureter	13.9	42	26	34
Other diseases of the genitourinary system	4.1	−27	−19	−9
**Complications of pregnancy, childbirth and puerperium**	0.1	−31	−22	3
**Certain conditions originating in the perinatal period**	1.8	7	6	2
**Congenital malformations and chromosomal abnormalities**	2.2	−41	−31	4
**Symptoms, signs, ill-defined causes**	58.4	253	194	15
**External causes of morbidity and mortality**	65.2	−40	−31	−13

MCW: Multiple-cause-of-death weighting method; AIDS: acquired immune deficiency syndrome; HIV: human immunodeficiency virus.

^a^ The cause-of-death categories and subcategories are those listed in the *European shortlist for causes of death, 2012*.^14^

^b^ With the classic method, standardized mortality was calculated using only the underlying cause of death specified on the death certificate.

^c^ Details of the three multiple-cause-of-death weighting methods, MCW_1_, MCW_2_ and MCW_3_, are given in the main text.

**Fig. 2:**
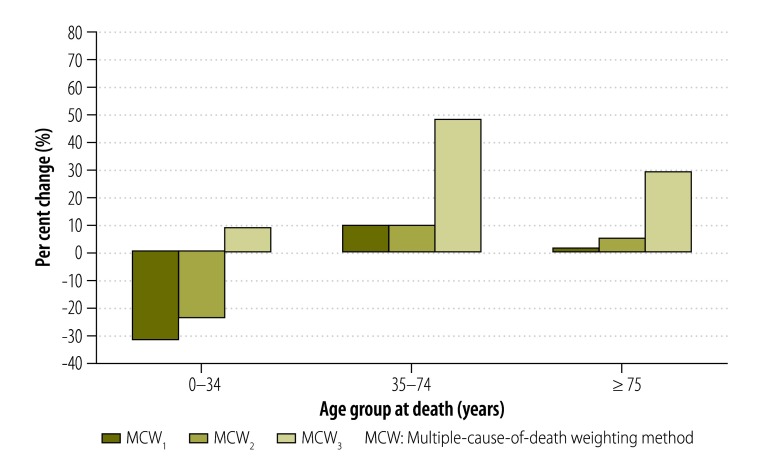
Change in standardized mortality for endocrine and nutritional diseases with multiple-cause-of-death weighting methods relative to the classic method, by age, 2010, France

**Fig. 3:**
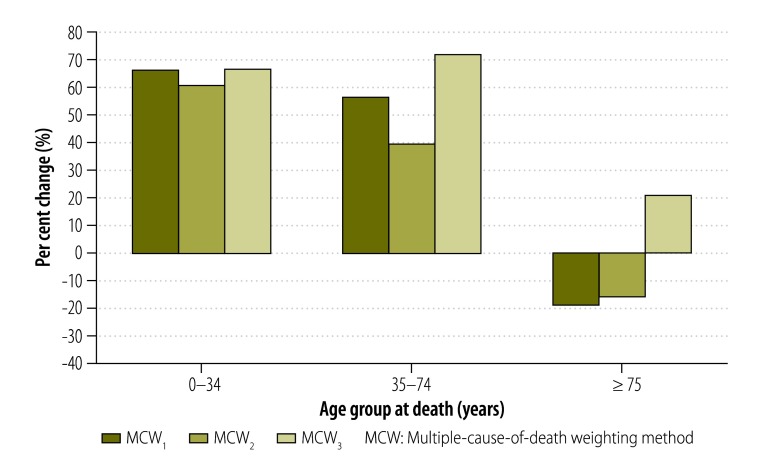
Change in standardized mortality for mental disorders with multiple-cause-of-death weighting methods relative to the classic method, by age, 2010, France

**Fig. 4:**
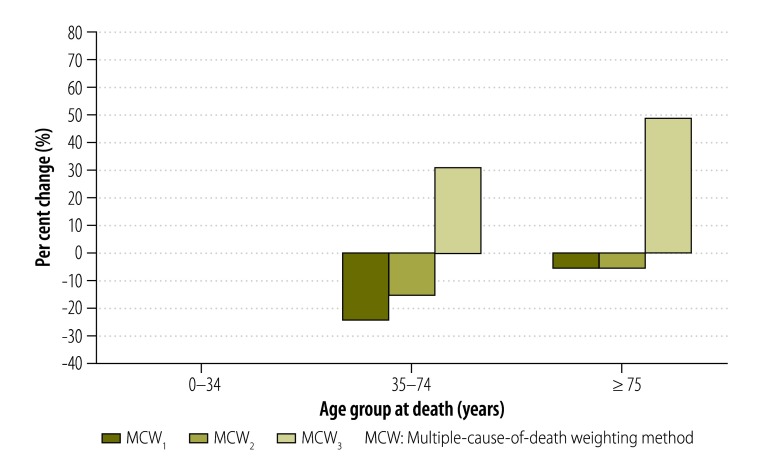
Change in standardized mortality for rheumatoid arthritis and osteoarthrosis with multiple-cause-of-death weighting methods relative to the classic method, by age, 2010, France

In addition, the MCW_3_ method also enabled us to highlight the increase in the mortality burden associated with certain conditions in specific age groups. For example, the increase in the standardized mortality rate derived using the MCW_3_ method relative to the classic method was as high as 48% for *endocrine and nutritional diseases* in people aged 60 to 69 years. The increase was very small in those aged 0 to 34 years, large in those aged 35 to 74 years and smaller again in those 75 years of age or older (Fig. 2). For *mental disorders*, the increase in mortality burden was much greater for people aged 0 to 34 years and 35 to 74 years than for those aged 75 years or older (Fig. 3). The increase in mortality burden for *rheumatoid arthritis and osteoarthrosis* was found to be greatest in people 75 years of age or older (Fig. 4).

MCW: Multiple-cause-of-death weighting method.

Notes: Details of the three multiple-cause-of-death weighting methods, MCW_1_, MCW_2_ and MCW_3_, are given in the main text. With the classic method, standardized mortality was calculated using only the underlying cause of death specified on the death certificate.

MCW: Multiple-cause-of-death weighting method.

Notes: Details of the three multiple-cause-of-death weighting methods, MCW_1_, MCW_2_ and MCW_3_, are given in the main text. With the classic method, standardized mortality was calculated using only the underlying cause of death specified on the death certificate.

MCW: Multiple-cause-of-death weighting method.

Notes: Details of the three multiple-cause-of-death weighting methods, MCW_1_, MCW_2_ and MCW_3_, are given in the main text. With the classic method, standardized mortality was calculated using only the underlying cause of death specified on the death certificate.

Analysing mortality data by sex using the MCW_3_ method did not reveal any other increases in the mortality burden associated with particular conditions in addition to those already identified in the overall analysis. Similar increases were observed for men and for women with the MCW_3_ method relative to the classic method, except for *mental disorders*, where the increase was 40% in men and 27% in women and for *genitourinary diseases*, where it was 29% and 15%, respectively.

## Discussion

Our analysis of all death certificates in France for 2010, in which we used three multiple-cause-of-death weighting methods to derive standardized mortality rates, aimed to provide a better estimate of the actual causes of death than the classic method. In particular, we confirmed the findings of previous studies that some conditions that are rarely designated as the underlying cause of death actually make a substantial contribution to mortality: namely, diabetes,^3^^,^^17^^,^^18^ skin disease, blood disease^9^^,^^19^ and renal disease.^1^^,^^3^^,^^7^ However, as previously observed,^3^ the increase in the standardized mortality rate we found for each condition varied widely with the disease category. In contrast, other conditions that we revealed to have contributed more to mortality than previously recognized were little mentioned in the literature, such as mental disorders^12^ and diseases of the musculoskeletal system, especially rheumatoid arthritis and osteoarthrosis.^20^ Moreover, application of the MCW_3_ method showed that the contribution of certain conditions to mortality varied even in young people: in particular, mental disorders contributed more in young people than indicated by the classic method. The contribution of conditions in other disease categories, such as *diseases of the circulatory system*, was found to be unaffected, or only slightly affected, by application of the MCW_3_ method, which again confirmed literature findings.^3^ In contrast to published results,^3^ we found that the contribution to mortality of some conditions, for example influenza, was less than indicated by the classic method. In particular, the contribution of conditions in the category *external causes of death* was much less. Although this finding may be surprising at first, it reflects the possibility that, even when the underlying cause of death was categorized as an external cause of death, the physician thought some other condition contributed to the death and chose to mention it on the death certificate.

One limitation shared by all studies on multiple causes of death is that data quality and comparability are not perfect and numerous studies have tried to identify the flaws.^21^^–^^25^ In addition, the numerous coding rules and the multiplicity and complexity of possible disease combinations listed on a death certificate could lead to misinterpretations. Nevertheless, mortality databases are essential for monitoring public health and all attempts to improve their use should be welcomed, especially those taking into account multiple causes of death. The weighting approach described in our study could help clarify the impact of various conditions on mortality in countries that collect multiple-cause-of-death data. For other countries, the existence of weighting methods could encourage a more systematic approach to the collection of data on multiple causes of death.

Another limitation is that the MCW_3_ method takes into account only the contributing causes of death mentioned in part II of the death certificate (in addition to the underlying cause) that are regarded as being on different causal pathways from the main morbid process. However, this assumption is correct only if the death certificate is properly completed, which may not be certain. Moreover, some information is lost by not attributing weights to all causes of death listed on part I. The MCW_3_ method may be less appropriate when the research question concerns a complication of a disease rather than the disease itself. Furthermore, when researchers are investigating a specific topic, the set of disease codes considered when implementing a weighting method can be adapted: for example, a study on the external causes of death could include ICD-10 cause-of-death codes that refer to types of injury or poisoning (i.e. codes beginning with S and T), which were excluded in the present study. Although we studied standardized mortality rates, the weighting method could also be applied in other ways. For instance, some policy-makers may be more interested in the crude number of deaths.

To date, we have not estimated the statistical variance of the indicators obtained using a weighting method. This may be a problem if a study is comparing mortality distributions between, for instance, several locations. One solution would be to use a nonparametric bootstrap approach. However, as our analysis considered a large number of deaths, sampling variability should not affect the interpretation of the results.

The main limitation of our study is that the process of weighting multiple causes of death provides only a synthetic view of the causal process by which diseases act together to bring about death.^13^ Consequently, the values given to the weights are subjective and weighting methods could be used to carry out a sensitivity analysis that takes into account different possibilities. In the future, the assignment of weights to items listed on a death certificate could be done by international consensus. Research is needed to determine the value of the weights that should be attributed to the different causes of death contributing to a death, although this process may also be based on a subjective view of how causal responsibility is distributed among different causes of death.^26^ Further, this process would require large longitudinal databases that record pathological conditions and health events over time. Finally, it would be useful to have international rules that assign a specific role to each cause of death mentioned on a death certificate. In particular, the weight given to ill-defined causes of death and cardiac arrest should probably be smaller than that given to other causes. These international rules could also help to systematically distinguish causes of death on separate causal pathways. Moreover, death certification by physicians should be standardized both within and between countries to improve the comparability of the statistics obtained.

In conclusion, although it is valuable to know the underlying cause of death, the contribution of other possible causes of death listed on a death certificate should not be neglected. The multiple-cause-of-death weighting methods we used in this study to assess the contribution of different conditions to mortality are promising. Previously, we applied a similar weighting approach to study the burden of mortality, and the etiological processes, associated with individual diseases using survival regression models.^13^
